# Norwegian Orthodontists’ Experience and Challenges With Treatment of Patients With Cleft Lip and Palate

**DOI:** 10.1177/10556656211028509

**Published:** 2021-07-20

**Authors:** Paul K. Saele, Anne-Kristine Nordrehaug Aastrøm, Harald Gjengedal, Elwalid F Nasir, Manal Mustafa

**Affiliations:** 1 Oral Health Centre of Expertise/Western Norway, Department of Clinical Dentistry, University of Bergen, Norway; 2 Department of Clinical Dentistry, University of Bergen, Norway; 3 Department of Clinical Dentistry, University of Bergen, Norway; 4 King Faisal University SA, University of Science and Technology, Omdurman, Sudan; 5 Oral Health Centre of Expertise/Western Norway, Bergen, Norway

**Keywords:** CL/P, orthodontist, experience, education, Norway

## Abstract

**Background:**

Patients born with cleft lip and/or palate (CL/P) have orthodontic treatment challenges due to maxilla deficiency, malocclusions, and dental abnormalities. In Norway, orthodontic treatment is done by centralized CL/P teams. Due to traveling restrictions, this treatment might be done locally in the future. The experience of Norwegian community orthodontists in managing such patients has not been investigated previously.

**Objective:**

To assess Norwegian orthodontists’ management of patients with CL/P and need for further education.

**Material and Methods:**

All orthodontists in Norway were sent a questionnaire about their experience, challenges, and knowledge and asked about their need of further theoretical education and clinical training in the management of patients with CL/P.

**Results:**

Norwegian orthodontists’ standard of knowledge of CL/P treatment is adequate. However, few respondents have treated a high number of cleft patients. Eighty-six percent of the participants believed that treating CL/P patients involves challenges, such as time-consuming treatment and technical difficulties. Increased perceived need for more education was revealed among participants stated unpreparedness during education (4 folds), encountered challenges, and lack of knowledge (almost 3 folds).

**Conclusions:**

The study revealed that community orthodontists in Norway lack experience and acknowledged the challenges in treating patients with CL/P. Most of the respondents perceived a need for additional education and clinical training to treat CL/P patients competently. The findings suggested more focus on patients with CL/P management in the curricula and more collaboration between centralized CL/P teams and community orthodontists.

## Introduction

Orofacial clefts are considered to be the most common craniofacial anomaly (Vanderas, 1987; Sayetta, 1990), with a wide range of individual facial and dental abnormalities (Pegelow et al., 2012; Brignardello-Petersen, 2017). The typical cleft patient has a maxillary deficiency, resulting in malocclusions (Tindlund, 1994; DeLuke et al., 1997) and dental anomalies, such as agenesis, supernumerary teeth, or peg-shaped teeth (Tereza et al., 2010; Pegelow et al., 2012; Saele et al., 2017; Rizell et al., 2020). In addition to the anatomical abnormalities, patients with cleft lip and/or palate (CL/P) may have cognitive conditions that could challenge the delivery of dental care by the dentist/orthodontist (Feragen & Stock, 2014).

### Interdisciplinary Treatment

Patients born with CL/P require treatment by different medical specialists, and the outcome could be improved by organized interdisciplinary medical teams treating a high volume of patients (Semb et al., 2005; Shaw et al., 2005; Khavanin et al., 2019). “The Eurocleft project” by Semb and Shaw (Semb et al., 2005; Shaw et al., 2005) from 2005 concluded that standardization, centralization, and the participation of high-volume operators were associated with good treatment outcomes. Little information is available about cleft teams worldwide, and there is still no international consensus as to the preferred overall treatment (Kuijpers-Jagtman, 2006; Tindlund et al., 2009; Austin et al., 2010).

### The Role of the CL/P Orthodontist

The orthodontist should have adequate knowledge of the typical dental/orthodontic CL/P diagnosis, treatment options, and the timing of bone graft according to various cleft centers guidelines (Abyholm et al., 1981; Bergland et al., 1986; Bergland et al., 1986; Semb et al., 1986; Williams et al., 2003; Batra et al., 2004; Russell et al., 2016), or orthognathic surgery (DeLuke et al., 1997). Increasing the frequency of treating patients with CL/P would give the orthodontist clinical experience, and the collection of patient records would provide valuable data for retrospective studies (Shaw et al., 2001; Shaw et al., 2005).

### Education and Training of Orthodontists

Specialist training of orthodontists should include consideration of patients with CL/P as a group with special needs. In a study by Lewis et al. (2005) from Washington, 156 orthodontists reported lack of knowledge and experience of orthodontic care of patients with CL/P. Similar findings are reported in later studies from Korea, the United States, and Canada (Noble et al., 2010; Cho et al., 2012). These results support the need for additional education on management of CL/P patients at both under- and postgraduate levels, as well as through various courses and clinical training workshops for dental health personnel (Lewis et al., 2005; Noble et al., 2010; Cho et al., 2012).

### The Norwegian CL/P System

The incidence of CL/P in Norway is 1.8 in 1000 live births annually (Abyholm, 1978). All patients are treated by one of the 2 national interdisciplinary teams of medical specialists. As in many other international CL/P teams, the orthodontist plays a pivotal role in patient management (Shaw & Semb, 1990; Kuijpers-Jagtman, 2006). Most of the active orthodontic treatment is done at the centers, and the frequency of appointments is determined by the severity of the malformation and the extent of the treatment. All expenses are covered by the Norwegian government.

The burden of traveling long distances, in addition to pandemic restrictions, constitutes challenges for providing orthodontic treatment by centralized CL/P teams in Norway. These demanding conditions might call for a more active role for community orthodontists in the future. Therefore, education and calibration of the community orthodontist will be of increasing importance for the CL/P treatment outcome.

## Aim

This study focused on Norwegian orthodontists aiming to assess experience, challenges, knowledge, and self-perceived need for further theoretical education and practical training related to management of patients with CL/P.

## Material and Methods

A letter about informed consent and a questionnaire were sent electronically to all 275 members of the Norwegian Orthodontic Society. SurveyXact by Ramboll (Surveyxact.com) distributed the questionnaires and collected the data between October 2019 and January 2020. The following participants were excluded from the study group: 57 of the orthodontists who replied that they were retired, 39 who did not respond, 6 who are involved in the centralized CL/P teams, and 2 of the respondents who had taken part in the pilot study. This gave a response rate of 78.4% (171/218).

### Demographic Information

Demographic information included gender and age (under 50 or 50 years and older). Data were collected about country/city of dental and orthodontic education: Norway, Scandinavia other than Norway, Europe other than Scandinavia, and outside Europe. Information was collected as type of practice: private or public orthodontic clinics and university employment, with or without clinical duties.

### Experience and Possible Challenges

We assessed information regarding orthodontists’ experience including (a) years since graduation as a dentist (less than 30 or 30 years and more), (b) years of experience as an orthodontist (less than 20 or 20 years and more), (c) total number of orthodontic patients treated per year (fewer than 200 or 200 and more) and (d) total number of cleft cases treated during their career (fewer than 10 or 10 and more). An additive sum score was constructed from the 4 items (a, b, c, and d), with a response score ranging from 0 to 4. Scores 1 to 2 are considered to be low experience and 3 to 4 to be high.

All respondents were asked about possible challenges they might have experienced in treating CL/P patients compared to patients without clefts. The answers were scored as yes (1) for the presence of challenges and no/I do not know (0) for the absence of challenges. The respondents were further asked about different challenges (technical, theoretical, time-consuming treatment, emotional challenges, and problems with compliance that the orthodontist might encounter while treating CL/P patients): The response was either yes (1) or no (0). The respondents were further asked for written comments and examples of the type of challenges they might have encountered.

### Knowledge

Four items were used to measure knowledge. The orthodontists were asked about (a) development of a cleft, (b) the different dental abnormalities, (c) malocclusions that are specific to this group of patients, and (d) whether these patients have additional medical problems compared to those without a cleft. The answers were scored as yes (1) for good knowledge and no/I do not know (0) for poor knowledge. An additive sum score was constructed from the above 4 items (a, b, c, and d), with a response score ranging from 0 to 4. Familiarity with the Norwegian CL/P system was explored, as well as the frequency of contact with centralized cleft teams or other health authorities.

### Education and Clinical Training

The questionnaire further explored whether undergraduate and/or postgraduate education prepared the community orthodontist to manage patients with CL/P. The study also investigated the orthodontists’ perceived need for more theoretical education or clinical training in the treatment of this group of patients. The participants responded to the questions using a Likert scale, ranging from strongly agree (1), agree (2), do not know (3), disagree (4), to strongly disagree (5). The variables were further dichotomized into agree (1), combining the initial categories 1 and 2, and disagree (2), combining the initial categories 3 to 5. The need for both theoretical and clinical education was further dichotomized to 1 (need) and 0 (no need), and the sum of both theoretical and clinical need was scored, ranging from 0 to 2.

### Data Analysis

The data were analyzed using SPSS version 24 (SPSS Inc). Descriptive statistics including frequency and percentages of categorical variables were calculated and tabulated. Bivariate analysis was performed using cross tabulation and the χ^2^ test exploring the association of the dependent variable, “need for theoretical education and clinical training,” with the independent variables “experience, clinical settings, and challenges.” A 2-sided significance level of 5% was implied for all analyses. Multiple variable analysis was performed with a binary variable as the outcome, using logistic regression and estimating odds ratio (OR) and 95% CIs.

### Ethical and Legal Considerations

Ethical approval was granted by the local legal authorities of Norwegian Social Science Data Services (NSD in 2017, id# 53108). All data are processed without a name, and a code number links these data through a list of names. It will not be possible to identify the participants from the results of the published study. The study was funded by Vestland County, Oral Health Centre of Expertise.

## Results

### Study Profile

The distribution of the participants’ sociodemographic characteristics according to gender is presented in the supplementary file. More than half of the respondents were males (55.6%), and 52.6% were 50 years or older. However, in the age group under 50, females predominated. Most had undertaken their specialist education in orthodontics in Norway (81.3%) or another Scandinavian country (11.1%). Most were in private practice (91.2%), either full time or part time. A limited number of respondents (n = 15) practice orthodontics only in public service or at one of the 3 universities in Norway (data presented in supplementary file).

### Experience

Table 1 presents the participants’ experience as a dentist/orthodontist treating patients with and without CL/P in total and according to gender. A larger portion of males (49.5%) than females (34.2%) reported longer experience (>30 years) as dentists. The corresponding figures for participants with more than 20 years’ experience as orthodontists were 51.6% and 32.9%, respectively. More male than female orthodontists reported seeing over 200 new orthodontic patients in their clinic each year and having seen more CL/P patients during their careers (Table 1).

**Table 1.** Participants’ Work Experience by Gender (n = 171).

Variable	Male, % (n)	Female, % (n)	Total, % (n)
Experience			
<30 years as a dentist	50.5 (48)	65.8 (50)	57.3 (98)
≥30 years as a dentist	49.5 (47)	34.2 (26)^a^	42.7 (73)
<20 years as an orthodontist	48.4 (46)	67.1 (51)	56.7 (97)
≥20 years as an orthodontist	51.6 (49)	32.9 (25)^a^	43.3 (74)
Ortho patients/year			
<200	38.9 (37)	50.7 (38)	44.1 (75)
≥200	61.1 (58)	49.3 (37)	55.0 (95)
CL/P patients treated/career			
<10	47.4 (45)	56.6 (43)	51.5 (88)
≥10	52.6 (50)	43.4 (33)	48.5 (83)
Sum of work experience			
Low	26.3 (25)	46.7 (35)	35.3 (60)
High	73.7 (70)	53.3 (40)^b^	64.7 (110)

Abbreviation: CL/P, cleft lip and/or palate.

^a^ *p<*0.05.

^b^ *p<*0.001.

Data showed that half the Norwegian orthodontists (51.8%) see between 150 and 299 new orthodontic patients each year (data not presented in table 1). Most of the respondents (63.7%) reported having treated a limited number (fewer than 20) of CL/P patients during their careers, and 13.5% reported that they had never treated a CL/P patient. Only 2.4% of the community Norwegian orthodontists reported that they had treated more than 100 CL/P patients during their careers. The results showed that 148 responding orthodontists (86.5%) treated CL/P patients with different orthodontic appliances, mainly fixed appliances in the permanent dentition (97%), but a few reported treatments during the mixed dentition stage (28.4%).

### Challenges

Table 2 reflects the challenges encountered in treating cleft patients. Eighty-six percent of the study participants were of the opinion that treating patients with CL/P might present different challenges from those encountered in treating patients without a cleft. Most of the participants (86.5%) have treated CL/P patients and they reported various challenges, such as time-consuming treatment (61.5%) and technical challenges (44.6%). The study did not show any statistical differences between challenge scores and the respondent’s gender, age, place of work, or the number of patients the respondent is treating each year (data not shown). Notably, all respondents were aware of the presence of centralized cleft teams in Norway and 80% of the respondents reported that they would consult the team, while 10% would seek advice from a colleague when they encountered challenges during the treatment of patients with CL/P.

**Table 2.** Potential Challenges (n = 171) and Challenges the Responders Have Encountered (n = 148).

Challenges (yes)	Male, % (n)	Female, % (n)	Total, % (n)
Potential challenges (n = 171)	55.8 (82)	44.2 (65)	86 (147)
Response from orthodontists treating CL/P patients (n = 148)			
Time-consuming treatment	67 (61)	33 (30)	61.5 (91)
Technical challenges	50 (33)	50 (33)	44.6 (66)
Theoretical challenges	58.8 (10)	41.2 (7)	11.5 (17)
Problem with cooperation	48.9 (7)	61.1 (11)	12.2 (18)
Emotional challenges	36.4 (4)	63.6 (7)	7.4 (11)

Abbreviation: CL/P, cleft lip and/or palate.

### Knowledge

As depicted in Table 3, about 80% of the respondents considered that patients with CL/P have specific dental (78.7%) or orthodontic problems (81.5%), which differ from those in patients without a cleft. Forty-five percent of all respondents believed that patients with CL/P defects have additional medical problems. Seventy-seven percent of the respondents are familiar with the pathogenesis and development of a CL/P defect and can explain this to the patients and parents. No gender difference was observed with respect to knowledge about CL/P. According to the knowledge sum score, 31.5% of the study participants had good knowledge of all possible dental and medical problems in CL/P patients.

**Table 3.** Percentage of Participants With Good Knowledge of CL/P (n = 171).

Knowledge (yes)	Male, % (n)	Female, % (n)	Total, % (n)
I can manage to explain the development of CL/P to patients and parents.	78.9 (75)	74.7 (56)	77.1 (131)
I have knowledge about/special dental problems among CL/P patients	76.6 (72)	81.3 (61)	78.7 (133)
I have knowledge about special orthodontic problems among CL/P patients	78.5 (73)	85.3 (64)	81.5 (137)
I have knowledge about additional medical problems among CL/P patients	48.9 (46)	40.5 (30)	45.2 (76)
Sum of knowledge (good)	29.7 (27)	33.8 (25)	31.5 (52)

Abbreviation: CL/P, cleft lip and/or palate.

### Preparedness for Treatment and Need for Further Theoretical and Clinical Education

The gender distribution of the respondents’ self-reported challenges and perceived need for further theoretical training are presented in Table 4. Most of the participants (87.7%) reported that their undergraduate education did not prepare them well for treating patients with orofacial clefts. However, only about 40% stated that they were not prepared for the treatment of CL/P after postgraduate orthodontic studies. Respondents stated that they had gained adequate knowledge after postgraduate studies, through participation in orthodontic congresses, courses, or reading the literature. Many orthodontists, however, reported a perceived need for additional theoretical education (78.1%) or clinical training (58.2%). A total of 55.0% reported a need for both theoretical and clinical training.

**Table 4.** Participants’ View of Whether Undergraduate and Postgraduate Education Prepared Them to Treat CL/P and Perceived Need for Further Theoretical and Clinical Education (n = 171).

Variable agree	Male, % (n)	Female, % (n)	Total, % (n)
Undergraduate education did not prepare me to treat CL/P	88.4 (84)	86.8 (66)	87.7 (150)
Postgraduate education did not prepare me to treat CL/P	36.8 (35)	43.4 (33)	39.8 (68)
Confirmed need for theoretical education	82.1 (78)	73.0 (54)	78.1 (132)
Confirmed need for clinical training	60.0 (57)	56.0 (42)	58.2 (99)
Confirmed need for both theoretical and clinical education	59.6 (56)	40.4 (38)	55.0 (94)

Abbreviation: CL/P, cleft lip and/or palate.

A confirmed need for further theoretical education, clinical training, and both clinical and theoretical education (total need) according to sociodemographics, work experience, knowledge, and challenges is presented in Table 5. The proportion of participants who confirmed a need for both theoretical and clinical training was significantly higher among those who reported that they were not prepared for the treatment of CL/P patients (91%), confirmed challenges (83.6%), and reported poor knowledge (86.6%) than among their counterparts who reported being prepared (73.8%), meeting no challenges (60.0%), and having good knowledge (69%). The need for further theoretical education was reported by a higher proportion of participants with poor knowledge than those with good knowledge (83.9% vs 67.3%, *P* < .05). The corresponding figures with respect to the need for clinical training were 63.7% and 48.1%, respectively (*P* < .05). Corresponding figures for participants who confirmed and disconfirmed challenges were 81.5% and 55.0%, respectively.

**Table 5.** Covariates of Participants’ Confirmed Need for Theoretical Education, Clinical Training, and Both Clinical and Theoretical Education.^a^

	Participants’ confirmed need for both theoretical education and clinical training, % (n)	Participants’ confirmed need for theoretical education, % (n)	Participants’ confirmed need for clinical training, % (n)
Age			
Under 50	83.8 (67)	82.5 (66)	60.5 (49)
50 years or more	77.5 (69)	72.4 (66)	56.2 (50)
Gender			
Male	83.2 (79)	82.1 (78)	60.0 (57)
Female	77.0 (57)	73.0 (54)	56.0 (42)
Place of postgraduate studies			
Scandinavia	79.6 (125)		
Outside Scandinavia	90.0 (10)		
Experience as orthodontist			
<20 years	83.3 (80)	78.3 (47)	56.7 (34)
≥20 years	76.7 (56)	77.8 (84)	59.6 (65)
Number of CL/P patients treated			
<10	81.6 (71)	78.2 (68)	60.9 (53)
≥10	79.3 (65)	78.0 (64)	55.4 (46)
Knowledge			
Good knowledge	69.2 (36)	67.3 (35)	48.1 (25)
Poor knowledge	86.6 (97)^b^	83.9 (94)^b^	63.7 (72)^b^
Challenges in CL/P treatment			
No	60.0 (12)	55.0 (11)	40.0 (8)
Yes	83.6 (122)^b^	81.5 (119)^b^	61.2 (90)
Preparedness during postgraduate education			
Prepared	73.8 (76)	72.8 (75)	49.5 (51)
Not prepared	90.9 (60)^c^	86.4 (57)^b^	71.6 (48)^c^

Abbreviation: CL/P, cleft lip and/or palate.

^a^ Unadjusted χ^2^ test (n = 171).

^b^ *p*<0.05.

^c^ *p*<0.001.

Table 6 presents the results of multiple logistic regression analysis, where the need for both theoretical and clinical education was regressed based on age, gender, knowledge, treatment challenges, and level of preparedness from undergraduate and specialist education. The data indicated that compared to participants with good knowledge, those with poor knowledge were more likely to report a need for further theoretical education and clinical training. Moreover, participants who were not prepared to treat CL/P patients were more likely to confirm a perceived need for further education than those prepared to treat CL/P patients. The corresponding ORs were 2.8 (95% CI: 1.2-6.7) and 3.7 (95% CI: 1.3-10.1).

**Table 6.** Need for Both Theoretical and Clinical Education Regressed on Age, Gender, Knowledge, Treatment Challenges, and Preparedness From Education.^a^

Variables	Total need, OR (95% CI)
Age	1.4 (0.5-3.4)
Gender	1.9 (0.8-4.5)
Knowledge	
Good knowledge	1
Poor knowledge	2.8 (1.2-6.7)
Challenges regarding CL/P treatment	
No challenges	1
Challenges	2.5 (0.8-7.6)
Preparedness during postgraduate education	
Prepared well to treat CL/P patients	1
Not prepared well to treat CL/P patients	3.7 (1.3-10.1)

Abbreviation: CL/P, cleft lip and/or palate.

^a^ Multiple variable logistic regression, odds ratio (OR), 95% CI (n = 171).

### Statements

The final self-reported statements have not been tabulated but reflect feedback from the respondents. The participants believed that CL/P patients presented more dental and occlusal problems, including transverse–sagittal deviations, agenesis, and inadequate alveolar bone, constituting specific treatment challenges. Further, they stated that syndromes, problems with compliance/concentration, and speech/breathing problems might require more time-consuming appointments for the treatment of patients with CL/P defects. The participants considered that these patients require more complicated orthodontic treatment and are at higher risk of relapse than orthodontic patients without a cleft.

According to the participants, treatment of patients with CL/P required more clinical consultations, frequent communication with the national CL/P team, and more interdisciplinary treatment planning compared with patients without a cleft. Reimbursement for CL/P treatment by the Norwegian state is based on a cost evaluation, but 55% of the community orthodontists consider that the reimbursement is not commensurate with the complexity of the orthodontic treatment being provided. Despite the different challenges and the cost issue, the respondents reported willingness to treat CL/P patients. Further, one respondent stated: “Without a cleft team to ask, I would have been lost,” and another stated: “I get confused and stressed by the interdisciplinary treatment needed for CL/P patients.” All the respondents supported the existing system of centralized national teams, with experienced orthodontists available for consultation when guidance is needed.

## Discussion

In the present study, most of the participants have treated CL/P patients, but only 50% reported having treated more than 10 cleft patients in their career. The respondents described various challenges, but their knowledge of management of CL/P patients was good. More than half the respondents group reported a need for further clinical training and theoretical education.

Most orthodontic management of patients with CL/P in Norway is undertaken by the centralized interdisciplinary teams. This explains the low level of experience among community orthodontists with respect to treatment of these patients. The results are in accordance with the findings by Lewis et al. (2005), that among orthodontists in Washington, only 20% of respondents had seen more than 3 patients with CL/P in the past 3 years. Similar findings are also reported in parallel studies among US and Canadian orthodontists (Noble et al., 2010) and in South Korea (Cho et al., 2012).

Most of the orthodontists reported treating CL/P patients with fixed appliances in the permanent dentition and few at the mixed dentition stage. The literature describes complicated CL/P treatment carried out between birth and early adolescence (Moore, 1986; Shaw & Semb, 1990), and at the permanent dentition stage, cleft patients are often considered as regular orthodontic patients. This is consistent with previous reports where management of specific part of cleft treatment is advised to be carried out by centralized CL/P teams (Kuijpers-Jagtman, 2006; Shaw & Semb, 1990)

In the present study, more male than female orthodontists reported longer experience and having seen more patients with CL/P in their careers. Nevertheless, among the younger orthodontists, females comprised a larger proportion of participants. This reflects a trend toward more females in the dental professions, as documented in a previous study (Haslach et al., 2018).

The participants reported technical, theoretical, and emotional challenges and compliance issues, leading to more time-consuming treatment. These challenges are described in previous studies where patients with CL/P have multiple dental abnormalities (Tereza et al., 2010) and malocclusions (DeLuke et al., 1997) requiring complicated treatment by interdisciplinary teams (Shaw & Semb, 1990; Kuijpers-Jagtman et al., 2015; Vig & Mercado, 2015). In a study among Norwegian CL/P patients, time-consuming treatment has been an issue because of additional psychological conditions that could affect patient compliance and clinical care (Feragen &d Stock, 2014). Although most orthodontist intended to complete orthodontic treatment in least amount of time, this is not possible when dealing with patients born with CL/P. Despite the challenging treatment and the time-consuming issues, the respondents in our study reported willingness to treat this group of patients. Furthermore, the participants supported the existing system of national centralized interdisciplinary teams with experienced orthodontists available for consultation when needed.

The present study indicates that Norwegian orthodontists’ knowledge about CL/P treatment is adequate. Most of the respondents were aware that patients with CL/P have more dental abnormalities and malocclusions than patients without a cleft. However, fewer than half the orthodontists were aware of other medical problems. This can be attributed to the traditional Norwegian system, whereby difficult CL/P cases with multiple challenges in combination with syndromes are always followed up by the centralized team and are not referred to local orthodontic clinics. This is in accordance with studies from other countries, reporting low attendance of children with oral clefts at orthodontic clinics (Lewis et al., 2005; Noble et al., 2010; Cho et al., 2012).

The respondents stated that orthodontic postgraduate studies had more focus on clefts than the undergraduate dental course. However, only 60% stated that they had been adequately educated to treat patients with CL/P and declared a need for more theoretical education and clinical training. Similar findings have been reported previously and indicate a need for more focus on the management of CL/P in the university curriculum (Lewis et al., 2005; Noble et al., 2010; Cho et al., 2012).

This study indicates that orthodontists with poor knowledge of CL/P were more likely to report a need for further education than those with better knowledge. Moreover, participants who were not prepared to treat patients with CL/P were more likely than their counterparts to perceive a need for more clinical training. These findings are in accordance with those of other studies related to management of CL/P patients (Lewis et al., 2005; Noble et al., 2010; Cho et al., 2012). It has been suggested that simulation training can allow cleft palate education to move from observational to competency-based learning (Raveendran, 2020). Future research should focus on ways to educate students and practitioners more effectively about relatively rare conditions and how to manage such cases.

In the Eurocleft study from 2005, Semb et al. (2005) documented a number of benefits of the multidisciplinary team approach to ensure that patients with CL/P have equal access to high-quality services. However, the inconvenience of traveling long distances, in addition to pandemic restrictions, are challenging factors in the treatment of CL/P patients by centralized teams. These demanding conditions might call for a more active role for community orthodontists to undertake cleft treatment in the future. Therefore, it is important to provide the community orthodontists with appropriate scientific education and clinical training to manage CL/P patients competently.

The high response rate among Norwegian orthodontists strengthens the findings of the present study. However, the few numbers of patients with CL/P treated by community orthodontists is considered as a limitation to measure the experience and challenges. Because of the low number of orthodontists in Norway, the results might not reflect the experience of orthodontists internationally but serve as a reference for studies under comparable conditions.

## Conclusion

The study revealed that community orthodontists have equitable knowledge, encountered challenges, lack experience, and perceived need for more education concerning patients with CL/P. Additional education and participation of residents in attending interdisciplinary staff meeting concerning treatment of patients with CL/P might improve the knowledge and enhance the orthodontist clinical competence.

We concluded that more focus on CL/P treatment in the undergraduate/postgraduate curricula and more collaboration between community orthodontists and central CL/P teams will improve the management of patients with CL/P.

## Supplemental Material

Supplemental Material, sj-pdf-1-cpc-10.1177_10556656211028509 - Norwegian Orthodontists’ Experience and Challenges With Treatment of Patients With Cleft Lip and Palate Click here for additional data file. Supplemental Material, sj-pdf-1-cpc-10.1177_10556656211028509 for Norwegian Orthodontists’ Experience and Challenges With Treatment of Patients With Cleft Lip and Palate by Paul K. Saele, Åstrøm Anne Nordrehaug, Gjengedal Harald, Nasir F. Elwalid and Mustafa Manal in The Cleft Palate-Craniofacial Journal
