# Supplementary material for: Norwegian Orthodontists’ Experience and Challenges With Treatment of Patients With Cleft Lip and Palate
Source: Cleft Palate Craniofac J. 2021 Jul 20;59(7):859–66. doi: 10.1177/10556656211028509 (PMC9260468; doi:10.1177/10556656211028509)
Supplement: Supplemental Material, sj-pdf-1-cpc-10.1177_10556656211028509 - Norwegian Orthodontists’ Experience and Challenges With Treatment of Patients With Cleft Lip and Palate [file sj-pdf-1-cpc-10.1177_10556656211028509.pdf]

Norwegian orthodontists experience and challenges with treatment of patients with cleft lip and palate.

Supplementary file: Sociodemographic characteristics of participants according to gender (n=171).

Variable	Male % (n)	Female % (n)	Total% (n)
**Gender**	55.6 (95)	44.4 (76)	100 (171)
**Age**			
< 50 years	21.6 (37)	25.7 (44)*	47.4 (81)
≥ 50 years	33.9 (58)*	18.7 (32)	52.6 (90)
**Undergraduate studies:**			
Norway	36.8(63)	27.5(47)	64.3(110)
Scandinavia except Norway	9.4(16)	5.3(9)	14.6(25)
Europe except Scandinavia	6.4(11)	9.9(17)	16.4(28)
Outside Europe	2.9(5)	1.2(2)	4.1(7)
**Postgraduate studies**			
Norway	49.1(84)	34.5 (59)	81.3(139)
Scandinavia except Norway	6.4(11)	4.6 (8)	11.1 (19)
outside Scandinavia	2.3 (4)	4.7 (8)	7.0(12)
**Work**			
Private clinic	51.5 (88)	39.8 (68)	91.2 (156)
Not private clinic	4.1 (7)	4.7 (8)	8.8 (15)

**p<0.001, *p<0.05
